# Editorial: Mechanisms of Lung Fibrosis: Is Immunity Back in the Game?

**DOI:** 10.3389/fimmu.2022.882979

**Published:** 2022-03-17

**Authors:** Antoine Froidure, Katerina Antoniou, Marialuisa Bocchino, Enrico Conte

**Affiliations:** ^1^ Service de pneumologie, Cliniques Universitaires Saint-Luc, Bruxelles et Institut de Recherche Expérimentale et Clinique, UCLouvain, Bruxelles, Belgium; ^2^ Laboratory of Molecular & Cellular Pneumonology, Department of Respiratory Medicine, School of Medicine, University of Crete, Heraklion, Greece; ^3^ Respiratory Medicine Section, Department of Clinical Medicine and Surgery, Federico II University, Naples, Italy; ^4^ Laboratory of Molecular Surfaces and Nanotechnologies (LAMSUN), University of Catania, Catania, Italy

**Keywords:** lung fibrosis, inflammation, immunity, inflammasome, fibrogenesis

Pulmonary fibrosis is a common feature to many interstitial lung diseases (ILDs). Among these, idiopathic pulmonary fibrosis (IPF) represents the paradigm of a “pure fibrotic” condition characterized by an intrinsic aggressiveness with inexorable evolution. Nevertheless, inflammation-driven ILDs, including for example connective tissue disease-associated ILDs (CTD-ILDs), sarcoidosis, chronic hypersensitivity pneumonitis, desquamative interstitial pneumonitis (DIP), can acquire a fibrosing phenotype which may lead to an accelerated lung function decline and tissue distortion, similarly to IPF (1). This clinical scenario, along with the discovery of common pro-fibrotic pathways, has led to the most recent concept of progressive fibrosing ILDs (PF-ILDs) (2). These entities must be carefully intercepted due to their rapidly evolving clinical behavior and unresponsiveness to conventional anti-inflammatory and immune-suppressive drugs. Following the failure of the PANTHER trial (3), the role of the immune system in the pathogenesis of fibrotic lung diseases has been minimized. Interestingly, recent data reveal that the subtle interplay between immune dysregulation and fibrogenesis produces a converging effect that may ultimately lead to end-stage fibrosis. On top of that, there is wide evidence that the natural history of IPF, and potentially of any other ILD, may be diverted from the interoccurrence of unpredictable acute exacerbations (AEs) displaying a strong and uncontrolled inflammation (4). In the last years, the availability of two anti-fibrotic drugs, i.e. nintedanib and pirfenidone, has improved the clinical management of IPF patients slowing disease progression and preventing both AEs and hospitalization (5, 6). Their use also in patients affected by PF-ILDs appears as an increasingly concrete perspective in clinical practice (7, 8). Nevertheless, any effort should be made to tidy up the puzzle of lung fibrogenesis with the primary aim to open the way to more innovative and hopefully resolving treatment opportunities.

Immune mechanisms that may trigger, enhance, or modulate the intricate process of lung fibrosis are the focus of the current Frontiers Research Topic. Original research and review articles cover cumulating evidence of the role of macrophages, monocytes, lymphocytes, and other immune cell types such as innate lymphoid cells and dendritic cells in the process of lung fibrosis. With specific reference to the Research Topic contents, Li et al. have shown that chronic hypoxia, a salient feature of pulmonary fibrosis, is able to trigger in IPF a specific immune response involving M1 and M0 macrophages, CD8 T cells, CD4 memory T cells, and mast cells. Very interestingly, the Authors have identified a hypoxia-immune-related gene signature that predicted disease prognosis. This signature, validated in 3 patient cohorts, is very promising in paving the way for future biomarkers. To demonstrate the close relationship between inflammation and fibrogenesis, Reynaud et al. have found that the loss of Club cells in the small airways of IPF patients was associated with aberrant regeneration processes. The data showed a not negligible relapse in clinical terms as lower SCGB1A1 expression, a Club cell marker, correlated with the extent of traction bronchiectasis on chest high-resolution computed tomography. Pro-fibrotic and pro-inflammatory profiles of airway macrophages (AMs) from IPF and PF-ILD patients were the focus of three additional original article contributions to the issue. Collagen-1a1 expression by AMs was described by Tsitoura et al. Profibrotic AMs co-expressing COL1A1 and OPN increased in the BAL of patients with IPF and other ILDs, while UIP pattern and a subsequent progressive phenotype were significantly associated with the higher BAL COL1A1 levels. Importantly, in non-IPF patients, higher COL1A1 levels were associated with a more than twofold increase in mortality. Additional data on the immune-inflammatory involvement in fibrogenesis comes from the observation that mitochondrial oxidation and alterations in bacterial burden in IPF and other ILDs may lead to augmented inflammasome activity in airway macrophages (AMs) (Jäger et al.). In this context, Trachalaki et al. have shown that NLRP3 was more inducible in IPF than in other ILDs in AMs, and that AIM2 inflammasome activation led to the expression of interleukin (IL)-1β, a key cytokine with both pro-inflammatory and pro-fibrotic properties. Whether this finding can be translated to explain the genesis of AEs remains elusive. Finally, inflammation-related pathways could constitute therapeutic targets outside “classical” immune-suppression. This is what was suggested by Steele et al. who showed that the TNF superfamily member 15 (TL1A) induced mucus production, inflammation and fibrosis through the expression of IL-13 by innate lymphoid cells. In a preclinical model of house dust mite-induced asthma, neutralization of TL1A by genetic deletion or antagonistic blockade of its receptor DR3 prevented all these events. This latter finding clearly illustrates how modulating inflammation could protect from fibrosis as in muco-secretory fibrotic diseases like severe asthma.

Additional contributions to this Frontiers Research Topic provide a quite extensive overview on current evidence of the involvement of frontline cell players like alveolar macrophages and epithelial cells in lung fibrogenesis. The interplay between the epithelium and immune cells is indeed able to significantly affect the lung microenvironment shaping a pro-fibrotic milieu. This picture may happen through the engagement of different pathways and mediators, as discussed in the papers by Plante-Bordeneuve and Froidure, and Kishore and Petrek, and is a prerequisite for the identification of promising therapeutic targets. Similarly, abnormal mucosal immunity along with the dysfunction and imbalance of dendritic cell subsets are intriguing and still poorly explored areas whose understanding will enrich our background of knowledge (Bocchino et al.). The role of regulatory immune cells was also reviewed (van Geffen et al.).

The commonalities of ARDS, COVID-19 and ILDs/IPF are also highlighted in this collection by Ntatsoulis et al. The authors emphasize on the increased levels of autotaxin (ATX) among the three syndromes and suggest that lysophosphatidic acid (LPA) signaling might be a shared pathogenetic pathway implicating amplified vascular damage, immune cell activation and promotion of fibrosis.

In conclusion, we believe that the characterization of the immune phenotype of IPF and fibrotic ILDs will help to discriminate endotypes with different clinical behavior thus allowing a personalized approach to the patient. In line with this expectation, Huaux proposes an interesting perspective on how we should interpret these findings and exploit them as attractive targets for future therapies. Our wish is that we may dispose in a not-too-distant time of increasingly effective treatments that can intercept and stop early aberrant fibrogenesis for the benefit of ILD patients.

## Author Contributions

All authors contributed to the redaction of the manuscript and approved its final version. AF and EC finalized redaction.

## Conflict of Interest

The authors declare that the research was conducted in the absence of any commercial or financial relationships that could be construed as a potential conflict of interest.

## Publisher’s Note

All claims expressed in this article are solely those of the authors and do not necessarily represent those of their affiliated organizations, or those of the publisher, the editors and the reviewers. Any product that may be evaluated in this article, or claim that may be made by its manufacturer, is not guaranteed or endorsed by the publisher.
